# Identification of Pathogenic Missense Mutations in the CHRNA5 Gene: A Computational Approach

**DOI:** 10.7759/cureus.47519

**Published:** 2023-10-23

**Authors:** Mahalakshmi Kumaraguru, Leelavathi L, Vijayashree J Priyadharsini, Meignana Arumugham I, Rajeshkumar S

**Affiliations:** 1 Public Health Dentistry, Saveetha Dental College & Hospital, Saveetha Institute of Medical and Technical Sciences, Saveetha University, Chennai, IND; 2 Clinical Genetics, Saveetha Dental College & Hospital, Saveetha Institute of Medical and Technical Sciences, Saveetha University, Chennai, IND; 3 Pharmacology, Saveetha Dental College & Hospital, Saveetha Institute of Medical and Technical Sciences, Saveetha University, Chennai, IND

**Keywords:** chrna5, genetics, genome-wide association study, smoking, nicotine dependence

## Abstract

Aim

The CHRNA5/A3/B4 gene locus is closely related to nicotine dependence and other smoking-related disorders. Coupling genetic and clinical studies of nicotine dependence and smoking behaviors may open new avenues for medication development. The aim of this study is to investigate the functional missense mutations in the CHRNA5 gene.

Methodology

The Ensembl database was used to gather data on missense mutations of the human CHRNA5 gene. Computational tools viz. SIFT (Sorting Intolerant From Tolerant), PolyPhen (Polymorphism Phenotyping), PROVEAN (Protein Variation Effect Analyzer), I-Mutant, and MutPred were used to uncover the pathogenic mutations in the gene under investigation.

Results

Among 161 missense variants reported inthe CHRNA5 gene, 94 variants were found to be highly pathogenic. Moreover, 20 were pathogenic and 4 were not pathogenic.

Conclusion

The computational analysis disclosed harmful mutations in* *the* *CHRNA5 gene which could be potentially associated with smoking-related traits.

## Introduction

Smoking increases the risk of lung cancer, heart disease, and chronic obstructive pulmonary disease [1]. Worldwide, countries have placed the reduction of smoking prevalence by early identification, prevention, and prompt treatment as their pre-eminent public health goal. Tobacco usage is propelled explicitly by nicotine dependence for a large part of smokers, considering that nicotine is the chief chemical culpable of tobacco addiction and reinforcement [2].

Hereditary influences contribute significantly to the process of nicotine dependence as has been proved with indisputable evidence [2]. Family and twin studies constitute the evidence of a high-intensity heritability of nicotine addiction [3,4]. Exhaustive genome-wide association study (GWAS) meta-analyses conducted recently, have indicated the importance of variation in the nAChR (nicotinic acetyl cholinergic receptor) subunit genes as the most vital genetic contributor to smoking behaviors [5,6].

A very crucial research area encompasses missense mutations, which are responsible for over half of all reported inherited diseases [7]. These are single nucleotide variants that result in amino acid substitutions at the protein level and are of two types, conservative and non-conservative (depending on the functional status of the protein) [8].

The most plausible authentication for smoking phenotypes such as the amount smoked (cigarettes per day, CPD), has been extricated from the CHRNA5-CHRNA3-CHRNB4 gene cluster situated on chromosome 15q25.1, which encodes for the subunits alpha5, alpha3, and beta4 [9]. On further scrutinization of the 15q25.1 region, the most well-established locus is indicated by the functional SNP rs16969968 which results in an amino acid change (D398N) in the alpha5 subunit, which in turn contributes to an increased nicotine intake by lowering the capacity of nAChRs to produce a timely inhibitory signal intended to control and limit nicotine consumption [10]. The link between the SNP rs16969968 in CHRNA5 and nicotine dependence was first noted in a candidate gene analysis done by Saccone SF et al. in 2007 [11].

Therefore, this study was formulated to identify potential functional mutations that may have a possible association with tobacco initiation, addiction, and cessation through the implementation of computational tools.

## Materials and methods

Data extraction

The rationale underlying the choice of CHRNA5 as the gene for this study is that genome-wide association studies have identified associations between the CHRNA5-CHRNA3-CHRNB4 gene cluster and smoking heaviness and nicotine dependence. Hence, the identification of pathogenic mutations in the CHRNA5 gene is an indispensable step to obtain an elaborate rendition of the pathogenesis of the gene. The FASTA sequence of the gene was downloaded from the National Center for Biotechnology Information website [12]. The data on missense mutations of the human CHRNA5 gene were collected from the Ensembl database [13]. As of June 2022, 161 missense mutations were identified and screened using three distinct computational tools which are SIFT (Sorting Intolerant From Tolerant), PolyPhen (Polymorphism Phenotyping), and PROVEAN (Protein Variation Effect Analyzer). The systematized data derived from the three software programs were subjected to further scrutiny using I-Mutant and MutPred to determine the stability of protein variants and their pathogenic potential respectively. Specifications regarding each of these software have been discussed in detail below.

Analysis using the SIFT program

An amino acid alteration is categorized by SIFT as either tolerated or deleterious to protein function. Intolerant/deleterious substitutions are those with a tolerance index less than 0.05 and those which are higher than 0.05 are classified as tolerated [14].

Analysis using the PolyPhen program

PolyPhen is a computerized program that predicts the potential effects of an amino acid substitution on a human protein's structure and function. A multitude of sequence, phylogenetic, and structural characteristics that describe the substitution are used to make the prediction. It then calculates the likelihood that the missense mutation will be damaging using an amalgamation of all these characteristics [15].

Analysis using the PROVEAN program

PROVEAN is a computer software program that determines if an amino acid substitution or indel will affect the biological functioning of a protein [16].

Analysis using the I-Mutant program

I-Mutant v3.0 is a tool that can forecast how a single-point mutation will impact the stability of a protein structure and how substantially a mutation in a protein sequence will or won't affect the stability of a folded protein [17].

Analysis using the MutPred program

MutPred2 is a tool that prioritizes pathogenic amino acid substitutions more effectively than current approaches, analyzes probable disease-causing molecular pathways, and provides decipherable pathogenicity score patterns on individual genomes. The chances that the mutation will have detrimental effects are identified [18].

## Results

**Figure 1:**
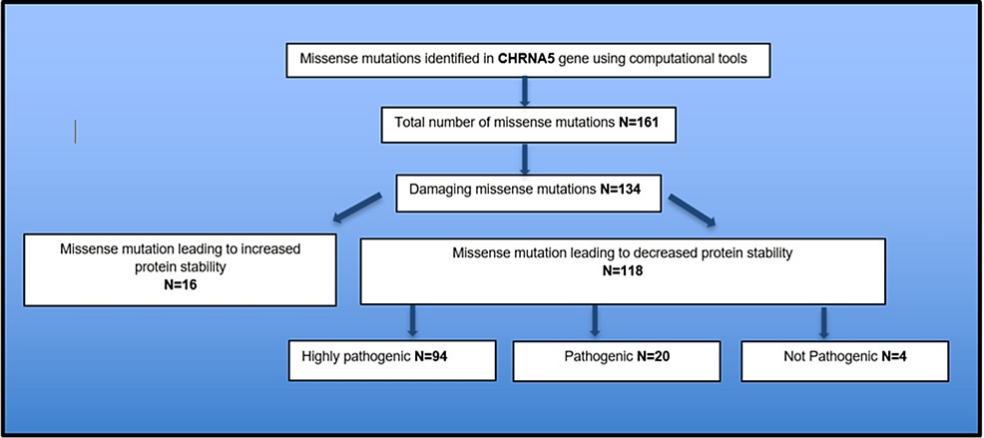
Schematic representation of the present investigation on the missense variants of CHRNA5 gene

Figure 1 represents a schematic view of the results of the study.

**Figure 2:**
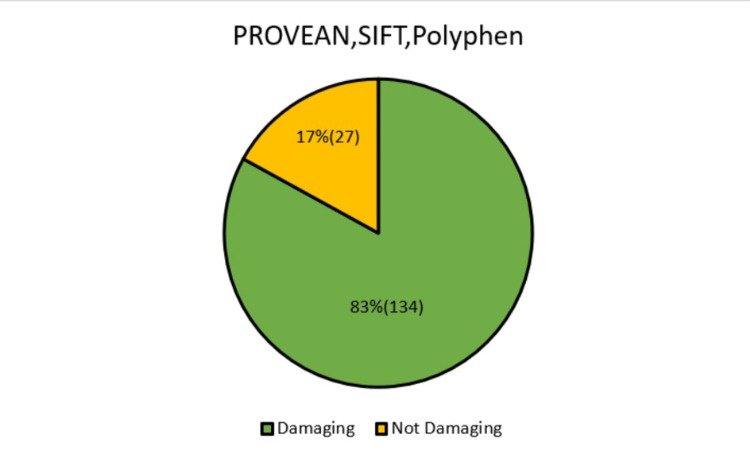
Percentage distribution of damaging and not damaging mutations as assessed by PROVEAN, SIFT, PolyPhen PROVEAN: Protein Variation Effect Analyzer, SIFT: Sorting Intolerant From Tolerant, PolyPhen: Polymorphism Phenotyping

The list of missense variants in the transcript (ENST00000299565.9) CHRNA5 gene classified on the basis of their influences as determined by the three gene prediction programs (SIFT, PolyPhen, and PROVEAN) were listed in a table (Appendix A). Out of 161 missense variants analyzed and screened, 134 SNPs were observed to possess a damaging potential as discerned by all three prediction computational tools elaborated in the methods column (Appendix A and Figure 2).

**Figure 3:**
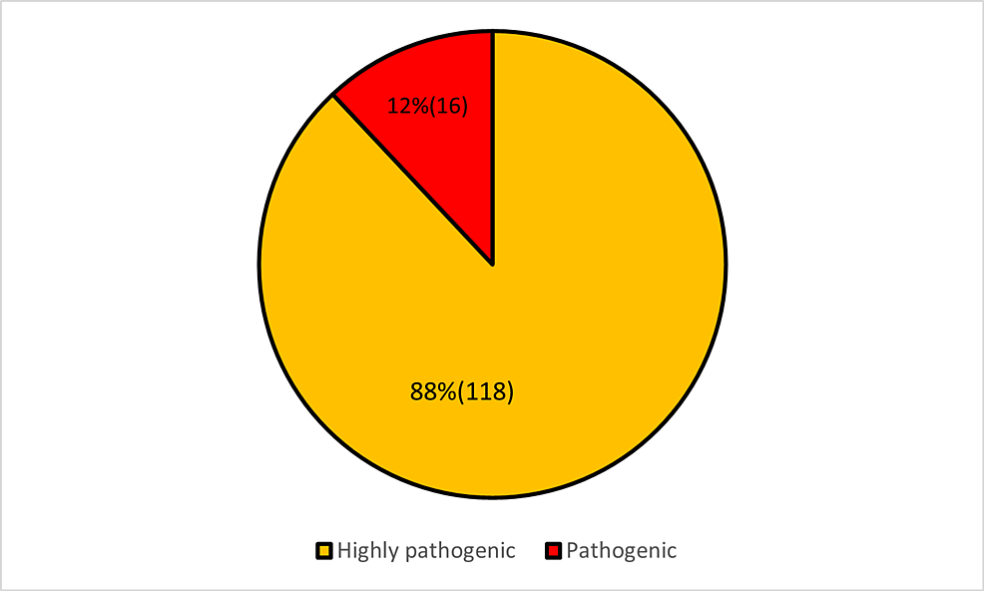
Percentage distribution of mutations with increased and decreased stability as assessed by I-Mutant

I-Mutant Suit software tool extricated 118 variants with a characteristic of decreased stability and 16 variants with increased stability (Appendix B and Figure 3).

**Figure 4:**
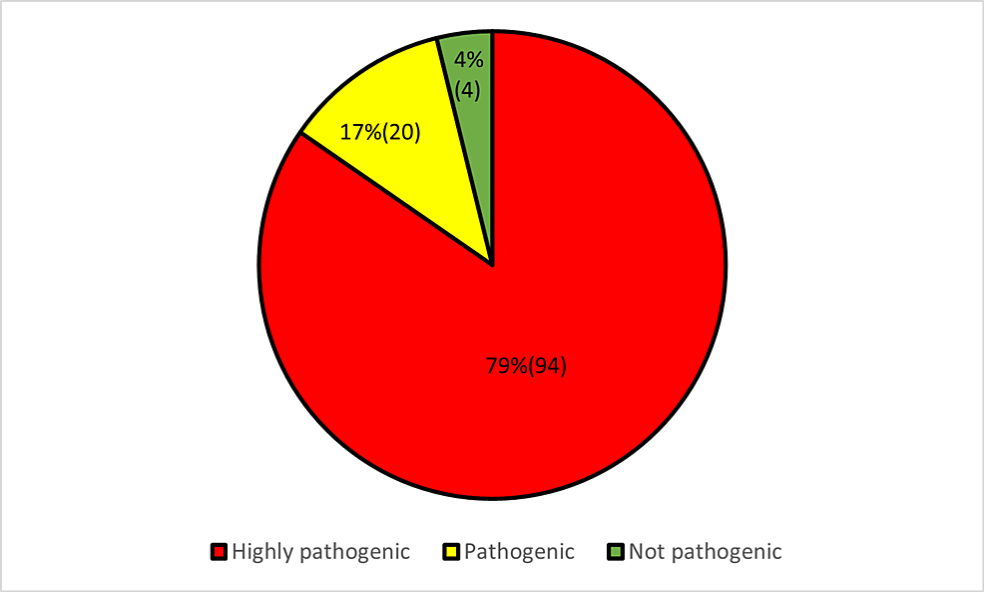
Percentage distribution of highly pathogenic, pathogenic, and not pathogenic mutations as assessed by MutPred

Moving forward, the MutPred software tool was employed to assess the pathogenicity of variants with decreased stability. From the 118 missense variants, 94 were observed to be highly pathogenic, 20 were observed to be pathogenic and 4 were observed to be not pathogenic with MutPred scores of >0.75, >0.5, and <0.5 respectively (Appendix C and Figure 4).

## Discussion

Nicotine dependency (ND) is a multifaceted condition with substantial rates of morbidity and mortality. According to Li et al., the projected heritability for ND is 0.46 in women and 0.59 in men, showing a sizable genetic influence [19].

For the identification of potentially harmful missense mutations, numerous computational methods have been invented which are also known as variant effect predictors. However, because different tools use distinct predictive traits, they frequently disagree with one another. Performance can be enhanced by ensemble approaches, which integrate the findings of numerous individual predictors [20].

Hence in this study, we used multiple computational tools such as SIFT, PolyPhen, PROVEAN, MutPred, and IMutant to identify pathogenic missense mutations in the CHRNA5 gene. These techniques use sequence information from homologs, structural information, like accessible surface area, and changes in amino acid properties to provide feature information as input to machine learning methods for phenotype prediction. They are conditioned on existing sets of mutation/phenotype association data [21].

In 2008, Berrettini et al. hypothesized that the CHRNA5/CHRNA3 genes that predispose a person to nicotine addiction create haplotypes, through their study of a European population [22]. Then, in 2009, Saccone et al. identified 11 SNPs in the CHRNA3 gene, one related SNP in the CHRNB4 gene, and five SNPs with substantial links with smoking in the CHRNA5 gene [23]. Al-Omoush et al. determined from a genomic DNA analysis of Jordanians that the rs16969968 SNP in the nAChR gene cluster CHRNA5-A3-B4 is strongly linked to waterpipe smoking dependency in Jordanians [24].

Genetic polymorphisms in CHRNA5 may increase the risk of nicotine dependency in Caucasian and African-American populations, according to a case-control study conducted by Sherva et al. [25].

Delving deeper, the relationship between smoking cessation and CHRNA5-A3-B4 gene variants was analyzed by researchers to result in conflicting conclusions. The results of a study done by Tyndale et al. exhibited an absence of association between CHRNA5-A3-B4 genetic variations/haplotype and post-treatment smoking cessation traits in Caucasian smokers by means of measuring the levels of cotinine which is the metabolite formed in the body after nicotine consumption, regardless of the high expected association between CHRNA5-A3-B4 genetic variants and pre-treatment smoking behaviors [26].

These observations are in consonance with previously conducted large genome-wide association studies which reported that smokers with CHRNA5-A3-B4 variations on chromosome 15 consume more tobacco than non-smokers but are not related to smoking status(i.e., current smoker vs. former smoker), demonstrating that these variants are not linked to smoking cessation [5]. However, in converse, Chen and coworkers and Bergen and coworkers found strong relationships between the CHRNA5-A3-B4 haplotype and quitting smoking in smokers who were administered a placebo treatment [27,28].

Projected fatalities from tobacco-related diseases are anticipated to ascend to unfathomable numbers in the coming years unless effective preventive measures are taken and prompt treatment can be provided. The utilization of genetics to enhance our current knowledge of the neurobiological mechanisms of nicotine is of prime importance in developing the foundational knowledge required for prompt action [29]. Hence, the utilization of software such as SIFT, PolyPhen, PROVEAN, I-Mutant, and MutPred can streamline the process of extricating genetic data as has been proven in previous literature, apart from saving time and manpower resources [30].

However, an important limitation of the study is the analysis of missense variants of only one transcript of the gene(ENST00000299565.9). Moreover, only missense mutations were analyzed in this study. Further elaborate studies taking into account all transcripts and other types of mutations are required to arrive at more irrefutable results.

The present in silico study was carried out to curate genetic variants from a large pool of single nucleotide variants reported in the CHRNA5 gene. Although there are many different types of variants, the missense variants were considered to be of utmost importance since they result in amino acid change, which eventually leads to structural or functional changes in the protein. Multiple tools were used to identify The tools such as SIFT, PolyPhen, and PROVEAN provide scores based on the substitution of amino acids. Those missense variants that were found to be associated with deleterious phenotype as ascertained by the 3 tools were further analyzed for protein stability using IMutant Suite. The majority of the variants were found to show decreased protein stability. Furthermore, these variants were checked for pathogenicity using another tool known as MutPred which predicts the impact of the genetic variants on protein functions based on 50 different protein properties. Thus, the present study provided us with 94 highly pathogenic variants, which can be further validated using experimental procedures to gain insight into their association with nicotine metabolism or smoking cessation.

## Conclusions

There have been considerable advances in decoding the role of genetics in nicotine dependence. However, inconsistent results and associations that are commonly small in magnitude pose a remarkable challenge to completely understanding genetic influences. Taking into account the above reports, this study has facilitated the process of extracting and organizing important preliminary data using an elaborate data extrication process to discern the potentially pathogenic variants of the CHRNA5 gene. This preliminary data will act as a useful tool and lay the foundation for further population-based studies that are warranted to conclude the association of these pathogenic variants with tobacco initiation, addiction, and cessation.

## Appendices

Appendix A 

**Table 1:** Catalogue of missense variants present in the transcript (ENST00000299565.9) of CHRNA5 gene classified on the basis of their effects as analyzed by three prediction software tools (SIFT, PolyPhen, and PROVEAN) PROVEAN: Protein Variation Effect Analyzer, SIFT: Sorting Intolerant From Tolerant, PolyPhen: Polymorphism Phenotyping

S. No.	Variant ID	AA	AA position	SIFT class	SIFT score	PolyPhen class	PolyPhen score	PROVEAN class	PROVEAN score
1	rs1449551218	L/F	50	deleterious	0	probably damaging	0.999	Deleterious	-3.682
2	rs200129149	Y/H	58	deleterious	0	probably damaging	1	Deleterious	-4.662
3	rs183719313	R/C	63	deleterious	0	probably damaging	1	Deleterious	-7.47
4	rs202057419	R/H	63	deleterious	0	probably damaging	0.999	Deleterious	-4.669
5	rs1300034813	P/L	64	deleterious	0	probably damaging	1	Deleterious	-9.338
6	rs56351164	F/V	76	deleterious	0	probably damaging	0.999	Deleterious	-5.721
7	rs778220199	F/Y	76	deleterious	0	probably damaging	0.998	Deleterious	-2.592
8	rs778220199	F/C	76	deleterious	0	probably damaging	0.999	Deleterious	-6.751
9	rs768348027	S/F	81	deleterious	0	probably damaging	0.95	Deleterious	-4.682
10	rs533238214	L/V	83	deleterious	0	probably damaging	0.997	Deleterious	-2.787
11	rs771858151	L/F	83	deleterious	0	probably damaging	1	Deleterious	-3.718
12	rs760430286	V/L	86	deleterious	0.03	probably damaging	0.981	Deleterious	-2.591
13	rs1344573389	V/A	86	deleterious	0	probably damaging	0.997	Deleterious	-3.629
14	rs1286786146	N/K	90	deleterious	0.01	probably damaging	0.999	Deleterious	-5.299
15	rs199649840	M/K	93	deleterious	0	probably damaging	0.993	Deleterious	-5.478
16	rs757754067	T/K	94	deleterious	0	probably damaging	0.999	Deleterious	-3.658
17	rs200691818	T/R	95	deleterious	0	probably damaging	1	Deleterious	-5.479
18	rs768366429	Q/R	101	deleterious	0	probably damaging	1	Deleterious	-2.893
19	rs768366429	Q/L	101	deleterious	0	probably damaging	1	Deleterious	-5.639
20	rs1182689078	W/C	103	deleterious	0.01	probably damaging	1	Deleterious	-11.57
21	rs1157428538	D/Y	105	deleterious	0	probably damaging	0.96	Deleterious	-8.133
22	rs1393443224	W/R	110	deleterious	0	probably damaging	1	Deleterious	-12.68
23	rs376394314	Y/C	115	deleterious	0	probably damaging	0.995	Deleterious	-7.683
24	rs754710874	G/E	117	deleterious	0	probably damaging	1	Deleterious	-5.487
25	rs1278994313	I/T	121	deleterious	0	probably damaging	1	Deleterious	-3.663
26	rs74913206	R/C	122	deleterious	0.01	probably damaging	1	Deleterious	-6.031
27	rs143659162	R/H	122	deleterious	0.03	probably damaging	1	Deleterious	-3.221
28	rs892335479	P/L	124	deleterious	0	probably damaging	1	Deleterious	-9.118
29	rs902937153	P/S	131	deleterious	0	probably damaging	0.999	Deleterious	-7.285
30	rs1180691964	D/E	132	deleterious	0	probably damaging	0.993	Deleterious	-3.646
31	rs2229961	V/F	134	deleterious	0	probably damaging	1	Deleterious	-4.591
32	rs765553764	R/C	142	deleterious	0.01	probably damaging	0.97	Deleterious	-3.67
33	rs1363250762	F/S	143	deleterious	0	probably damaging	0.999	Deleterious	-6.574
34	rs375192419	E/G	144	deleterious	0.03	probably damaging	0.999	Deleterious	-3.952
35	rs1237581945	T/I	157	deleterious	0.04	probably damaging	0.999	Deleterious	-2.81
36	rs55863434	P/L	163	deleterious	0	probably damaging	1	Deleterious	-9.482
37	rs200503890	Y/C	166	deleterious	0	probably damaging	1	Deleterious	-7.288
38	rs80087508	K/R	167	deleterious	0.01	probably damaging	0.998	Deleterious	-2.845
39	rs1259657704	S/R	168	deleterious	0	probably damaging	1	Deleterious	-4.741
40	rs1446367616	S/N	168	deleterious	0	probably damaging	0.994	Deleterious	-2.845
41	rs754212902	S/R	168	deleterious	0	probably damaging	1	Deleterious	-4.741
42	rs763840987	C/F	170	deleterious	0	probably damaging	1	Deleterious	-10.431
43	rs370096844	T/I	171	deleterious	0.05	probably damaging	0.999	Deleterious	-2.956
44	rs1567062384	V/A	174	deleterious	0	probably damaging	0.997	Deleterious	-3.789
45	rs780615731	T/M	175	deleterious	0	probably damaging	0.999	Deleterious	-4.317
46	rs1567062410	F/C	176	deleterious	0	probably damaging	0.999	Deleterious	-6.344
47	rs71528534	F/L	176	deleterious	0	probably damaging	0.996	Deleterious	-4.862
48	rs151059425	P/L	178	deleterious	0	probably damaging	1	Deleterious	-9.538
49	rs151206721	D/N	180	deleterious	0	probably damaging	0.999	Deleterious	-4.773
50	rs1383366253	D/E	180	deleterious	0	probably damaging	0.993	Deleterious	-3.819
51	rs1379297504	Q/R	182	deleterious	0	probably damaging	0.998	Deleterious	-3.819
52	rs1382179893	N/K	183	deleterious	0	probably damaging	0.999	Deleterious	-4.754
53	rs756066200	S/Y	185	deleterious	0.01	probably damaging	0.999	Deleterious	-3.446
54	rs779762437	M/V	186	deleterious	0	probably damaging	0.993	Deleterious	-3.524
55	rs1023953308	K/I	187	deleterious	0	probably damaging	0.999	Deleterious	-7.659
56	rs969761488	S/P	190	deleterious	0.01	probably damaging	0.999	Deleterious	-4.784
57	rs201173989	D/G	199	deleterious	0	probably damaging	1	Deleterious	-5.928
58	rs201173989	D/V	199	deleterious	0	probably damaging	1	Deleterious	-7.921
59	rs201956843	I/T	200	deleterious	0	probably damaging	0.981	Deleterious	-2.867
60	rs776966489	D/H	211	deleterious	0	probably damaging	1	Deleterious	-5.153
61	rs759969645	F/S	213	deleterious	0.02	probably damaging	0.999	Deleterious	-4.496
62	rs765530585	D/A	214	deleterious	0.01	probably damaging	1	Deleterious	-3.29
63	rs1483907535	N/D	215	deleterious	0	probably damaging	0.998	Deleterious	-2.526
64	rs761452231	G/R	216	deleterious	0	probably damaging	1	Deleterious	-6.075
65	rs1016759554	E/K	217	deleterious	0	probably damaging	0.997	Deleterious	-3.808
66	rs201346280	I/M	220	deleterious	0	probably damaging	0.999	Deleterious	-2.804
67	rs200252306	A/T	223	deleterious	0.04	probably damaging	1	Deleterious	-2.519
68	rs1219531112	G/R	225	deleterious	0	probably damaging	1	Deleterious	-6.643
69	rs747148242	P/S	238	deleterious	0.03	probably damaging	1	Deleterious	-4.52
70	rs61742337	P/R	238	deleterious	0	probably damaging	1	Deleterious	-5.701
71	rs61742337	P/L	238	deleterious	0	probably damaging	1	Deleterious	-3.897
72	rs1372147833	T/I	241	deleterious	0	probably damaging	1	Deleterious	-5.808
73	rs770212312	R/C	248	deleterious	0	probably damaging	0.999	Deleterious	-7.906
74	rs775819304	R/H	248	deleterious	0	probably damaging	0.999	Deleterious	-4.942
75	rs775819304	R/L	248	deleterious	0	probably damaging	0.999	Deleterious	-6.918
76	rs767124667	L/P	251	deleterious	0	probably damaging	1	Deleterious	-6.618
77	rs372825597	F/S	252	deleterious	0.01	probably damaging	0.997	Deleterious	-7.904
78	rs200232683	Y/C	253	deleterious	0.01	probably damaging	0.998	Deleterious	-8.886
79	rs200232683	Y/F	253	deleterious	0	probably damaging	0.983	Deleterious	-3.951
80	rs374468403	T/S	254	deleterious	0.03	probably damaging	0.955	Deleterious	-3.697
81	rs983271367	P/L	260	deleterious	0	probably damaging	1	Deleterious	-9.884
82	rs1309089415	C/R	261	deleterious	0	probably damaging	0.999	Deleterious	-11.466
83	rs1314435912	C/Y	261	deleterious	0	probably damaging	0.999	Deleterious	-10.544
84	rs201483179	I/N	262	deleterious	0	probably damaging	0.999	Deleterious	-5.195
85	rs201483179	I/S	262	deleterious	0	probably damaging	0.999	Deleterious	-4.133
86	rs868352551	S/L	265	deleterious	0	probably damaging	0.999	Deleterious	-5.635
87	rs138719535	F/I	266	deleterious	0	probably damaging	0.999	Deleterious	-4.494
88	rs926986572	Y/C	273	deleterious	0	probably damaging	1	Deleterious	-8.846
89	rs1596064105	L/R	274	deleterious	0	probably damaging	0.999	Deleterious	-5.918
90	rs936995586	P/S	275	deleterious	0	probably damaging	0.999	Deleterious	-7.871
91	rs770263622	E/D	280	deleterious	0	probably damaging	0.982	Deleterious	-2.949
92	rs749683576	C/R	285	deleterious	0	probably damaging	1	Deleterious	-9.852
93	rs1017054259	S/L	287	deleterious	0	probably damaging	0.999	Deleterious	-5.913
94	rs147498556	V/E	288	deleterious	0	probably damaging	0.999	Deleterious	-5.9
95	rs760176741	L/V	289	deleterious	0	probably damaging	0.993	Deleterious	-2.959
96	rs148560500	L/R	289	deleterious	0	probably damaging	0.999	Deleterious	-5.913
97	rs1487143659	L/S	292	deleterious	0	probably damaging	1	Deleterious	-5.633
98	rs200972466	T/S	293	deleterious	0	probably damaging	0.988	Deleterious	-3.936
99	rs765300879	F/Y	295	deleterious	0.03	probably damaging	0.997	Deleterious	-2.72
100	rs200010345	I/T	299	deleterious	0	probably damaging	0.999	Deleterious	-4.683
101	rs867154905	I/N	302	deleterious	0	probably damaging	1	Deleterious	-5.292
102	rs944978839	P/S	311	deleterious	0	probably damaging	0.999	Deleterious	-7.838
103	rs868388135	Y/C	316	deleterious	0	probably damaging	1	Deleterious	-8.785
104	rs116099178	L/V	317	deleterious	0.01	probably damaging	0.993	Deleterious	-2.943
105	rs74865777	M/V	321	deleterious	0	probably damaging	0.973	Deleterious	-3.926
106	rs200102110	V/L	324	deleterious	0	probably damaging	0.995	Deleterious	-2.869
107	rs867013198	T/I	331	deleterious	0	probably damaging	0.999	Deleterious	-5.901
108	rs1280180578	R/C	340	deleterious	0	probably damaging	0.999	Deleterious	-7.878
109	rs762980943	R/H	340	deleterious	0	probably damaging	0.999	Deleterious	-4.926
110	rs1567062983	H/R	345	deleterious	0	probably damaging	0.997	Deleterious	-6.651
111	rs1277622813	V/F	352	deleterious	0.01	probably damaging	0.951	Deleterious	-4.593
112	rs570647862	R/C	353	deleterious	0	probably damaging	1	Deleterious	-6.615
113	rs201385812	R/H	353	deleterious	0.05	probably damaging	1	Deleterious	-3.659
114	rs1567063057	L/F	360	deleterious	0.04	probably damaging	1	Deleterious	-3.093
115	rs1480979773	P/S	361	deleterious	0.01	probably damaging	1	Deleterious	-7.747
116	rs79109919	L/Q	363	deleterious	0	probably damaging	0.975	Deleterious	-3.739
117	rs1596064293	R/G	367	deleterious	0.01	probably damaging	0.999	Deleterious	-3.947
118	rs76766434	R/C	401	deleterious	0.01	probably damaging	0.91	Deleterious	-4.271
119	rs200127699	R/C	414	deleterious	0.02	probably damaging	0.91	Deleterious	-3.094
120	rs754450688	D/G	419	deleterious	0.01	probably damaging	0.984	Deleterious	-5.854
121	rs1327013276	W/C	420	deleterious	0	probably damaging	0.995	Deleterious	-11.26
122	rs934230114	K/Q	421	deleterious	0.01	probably damaging	0.999	Deleterious	-3.291
123	rs150329151	V/F	426	deleterious	0	probably damaging	0.999	Deleterious	-4.383
124	rs138253116	R/W	429	deleterious	0	probably damaging	1	Deleterious	-6.938
125	rs368612437	R/Q	429	deleterious	0	probably damaging	0.999	Deleterious	-3.463
126	rs776726741	W/G	433	deleterious	0	probably damaging	0.999	Deleterious	-10.058
127	rs1266274639	F/L	435	deleterious	0	probably damaging	0.996	Deleterious	-5.205
128	rs759802002	F/C	435	deleterious	0	probably damaging	0.999	Deleterious	-6.942
129	rs144352852	F/L	435	deleterious	0	probably damaging	0.996	Deleterious	-5.205
130	rs202066018	L/R	436	deleterious	0	probably damaging	1	Deleterious	-3.9
131	rs148376112	G/R	442	deleterious	0	probably damaging	1	Deleterious	-6.793
132	rs77773727	G/E	442	deleterious	0	probably damaging	1	Deleterious	-6.824
133	rs200384194	S/Y	443	deleterious	0	probably damaging	0.963	Deleterious	-3.167
134	rs532896027	L/P	446	deleterious	0	probably damaging	1	Deleterious	-5.238

Appendix B 

**Table 2:** Variants classified based on standard free energy change at 25°C with pH 7.0 after assessment of protein structural stability Variants that lead to increased stability of the protein (DDG > 0 – Increased stability; DDG < 0 – decreased stability)

S.NO	AA	AA position	stability	DDG (kcal/mol)
1	L/F	50	Increase	0.27
2	Y/H	58	Decrease	-1.85
3	R/C	63	Decrease	-1.66
4	R/H	63	Decrease	-2.84
5	P/L	64	Decrease	-0.27
6	F/V	76	Decrease	-2.57
7	F/Y	76	Decrease	-0.74
8	F/C	76	Decrease	-1.58
9	S/F	81	Decrease	-0.35
10	L/V	83	Decrease	-1.35
11	L/F	83	Decrease	-0.81
12	V/L	86	Decrease	-1.12
13	V/A	86	Decrease	-1.85
14	N/K	90	Decrease	-0.2
15	M/K	93	Decrease	-0.56
16	T/K	94	Decrease	-1.14
17	T/R	95	Decrease	-0.39
18	Q/R	101	Increase	0.24
19	Q/L	101	Increase	0.61
20	W/C	103	Decrease	-1.92
21	D/Y	105	Decrease	-0.89
22	W/R	110	Decrease	-2.06
23	Y/C	115	Increase	0.39
24	G/E	117	Decrease	-0.81
25	I/T	121	Decrease	-2.97
26	R/C	122	Decrease	-1.64
27	R/H	122	Decrease	-1.96
28	P/L	124	Decrease	-1.32
29	P/S	131	Decrease	-2.19
30	D/E	132	Decrease	-1.08
31	V/F	134	Decrease	-3.31
32	R/C	142	Decrease	-1.09
33	F/S	143	Decrease	-3.24
34	E/G	144	Decrease	-1.66
35	T/I	157	Decrease	-0.74
36	P/L	163	Decrease	-1.83
37	Y/C	166	Increase	0.85
38	K/R	167	Decrease	-0.54
39	S/R	168	Decrease	-1.05
40	S/N	168	Decrease	-1.12
41	S/R	168	Decrease	-1.05
42	C/F	170	Decrease	-1.09
43	T/I	171	Decrease	-0.75
44	V/A	174	Decrease	-3.17
45	T/M	175	Decrease	-0.32
46	F/C	176	Decrease	-2.39
47	F/L	176	Decrease	-2.78
48	P/L	178	Decrease	-0.57
49	D/N	180	Decrease	-0.7
50	D/E	180	Decrease	-0.23
51	Q/R	182	Decrease	-0.39
52	N/K	183	Decrease	-2
53	S/Y	185	Increase	0.11
54	M/V	186	Decrease	-0.65
55	K/I	187	Increase	0.15
56	S/P	190	Decrease	-1.93
57	D/G	199	Decrease	-0.92
58	D/V	199	Decrease	-1.87
59	I/T	200	Decrease	-3.05
60	D/H	211	Decrease	-1.79
61	F/S	213	Decrease	-2.98
62	D/A	214	Decrease	-2.97
63	N/D	215	Decrease	-1.13
64	G/R	216	Decrease	-2.35
65	E/K	217	Decrease	-2.5
66	I/M	220	Decrease	-0.96
67	A/T	223	Decrease	-0.88
68	G/R	225	Decrease	-1.82
69	P/S	238	Decrease	-2.78
70	P/R	238	Decrease	-1.52
71	P/L	238	Decrease	-1.49
72	T/I	241	Decrease	-0.17
73	R/C	248	Decrease	-2.19
74	R/H	248	Decrease	-2.09
75	R/L	248	Decrease	-1.06
76	L/P	251	Decrease	-1.41
77	F/S	252	Decrease	-1.82
78	Y/C	253	Increase	0.09
79	Y/F	253	Decrease	-0.79
80	T/S	254	Decrease	-0.27
81	P/L	260	Decrease	-1.93
82	C/R	261	Decrease	-0.83
83	C/Y	261	Increase	0.01
84	I/N	262	Decrease	-1.55
85	I/S	262	Decrease	-2.69
86	S/L	265	Decrease	-1.31
87	F/I	266	Decrease	-0.67
88	Y/C	273	Increase	0.04
89	L/R	274	Decrease	-2.17
90	P/S	275	Decrease	-1.77
91	E/D	280	Decrease	-0.45
92	C/R	285	Decrease	-0.98
93	S/L	287	Decrease	-0.8
94	V/E	288	Decrease	-1.88
95	L/V	289	Increase	0.12
96	L/R	289	Decrease	-1.76
97	L/S	292	Decrease	-2.04
98	T/S	293	Increase	0.04
99	F/Y	295	Decrease	-0.91
100	I/T	299	Decrease	-2.48
101	I/N	302	Decrease	-1.74
102	P/S	311	Decrease	-1.67
103	Y/C	316	Increase	0.73
104	L/V	317	Decrease	-0.89
105	M/V	321	Decrease	-0.76
106	V/L	324	Decrease	-1.07
107	T/I	331	Decrease	-1.13
108	R/C	340	Decrease	-0.75
109	R/H	340	Decrease	-1.6
110	H/R	345	Increase	0.08
111	V/F	352	Decrease	-3.24
112	R/C	353	Decrease	-0.89
113	R/H	353	Decrease	-1.26
114	L/F	360	Increase	0.61
115	P/S	361	Decrease	-0.62
116	L/Q	363	Decrease	-1.42
117	R/G	367	Decrease	-1.76
118	R/C	401	Decrease	-0.4
119	R/C	414	Decrease	-1.04
120	D/G	419	Decrease	-1.2
121	W/C	420	Decrease	-1.84
122	K/Q	421	Decrease	-1
123	V/F	426	Decrease	-2.26
124	R/W	429	Decrease	-0.9
125	R/Q	429	Decrease	-0.9
126	W/G	433	Decrease	-3.58
127	F/L	435	Decrease	-3.14
128	F/C	435	Decrease	-2.97
129	F/L	435	Decrease	-3.14
130	L/R	436	Decrease	-2.39
131	G/R	442	Decrease	-1.62
132	G/E	442	Increase	0
133	S/Y	443	Decrease	-1.35
134	L/P	446	Decrease	-1.57

Appendix C 

**Table 3:** Classification of pathogenicity of proteins with decreased stability as analyzed by MutPred tool Increased pathogenicity as predicted by MutPred (Score > 0.5 – pathogenic)

S.NO	Variant ID	AA	AA position	MutPred score
1	rs200129149	Y/H	58	0.849
2	rs183719313	R/C	63	0.87
3	rs202057419	R/H	63	0.791
4	rs1300034813	P/L	64	0.885
5	rs778220199	F/C	76	0.859
6	rs768348027	S/F	81	0.768
7	rs771858151	L/F	83	0.792
8	rs760430286	V/L	86	0.791
9	rs1344573389	V/A	86	0.824
10	rs1286786146	N/K	90	0.776
11	rs199649840	M/K	93	0.992
12	rs757754067	T/K	94	0.886
13	rs200691818	T/R	95	0.93
14	rs1182689078	W/C	103	0.964
15	rs1157428538	D/Y	105	0.922
16	rs1393443224	W/R	110	0.945
17	rs754710874	G/E	117	0.763
18	rs74913206	R/C	122	0.815
19	rs892335479	P/L	124	0.897
20	rs902937153	P/S	131	0.867
21	rs1180691964	D/E	132	0.811
22	rs2229961	V/F	134	0.89
23	rs1363250762	F/S	143	0.934
24	rs375192419	E/G	144	0.805
25	rs55863434	P/L	163	0.887
26	rs1259657704	S/R	168	0.929
27	rs1446367616	S/N	168	0.883
28	rs763840987	C/F	170	0.944
29	rs370096844	T/I	171	0.768
30	rs1567062384	V/A	174	0.881
31	rs780615731	T/M	175	0.763
32	rs1567062410	F/C	176	0.876
33	rs71528534	F/L	176	0.771
34	rs151059425	P/L	178	0.888
35	rs151206721	D/N	180	0.881
36	rs1383366253	D/E	180	0.859
37	rs1379297504	Q/R	182	0.927
38	rs1382179893	N/K	183	0.844
39	rs779762437	M/V	186	0.878
40	rs969761488	S/P	190	0.946
41	rs201173989	D/G	199	0.914
42	rs201173989	D/V	199	0.927
43	rs776966489	D/H	211	0.817
44	rs759969645	F/S	213	0.924
45	rs765530585	D/A	214	0.762
46	rs1483907535	N/D	215	0.844
47	rs761452231	G/R	216	0.924
48	rs1016759554	E/K	217	0.929
49	rs1219531112	G/R	225	0.932
50	rs747148242	P/S	238	0.779
51	rs61742337	P/R	238	0.885
52	rs61742337	P/L	238	0.814
53	rs1372147833	T/I	241	0.827
54	rs770212312	R/C	248	0.942
55	rs775819304	R/H	248	0.894
56	rs775819304	R/L	248	0.945
57	rs767124667	L/P	251	0.952
58	rs372825597	F/S	252	0.927
59	rs200232683	Y/F	253	0.777
60	rs983271367	P/L	260	0.838
61	rs1309089415	C/R	261	0.941
62	rs201483179	I/N	262	0.847
63	rs201483179	I/S	262	0.818
64	rs868352551	S/L	265	0.781
65	rs1596064105	L/R	274	0.91
66	rs936995586	P/S	275	0.854
67	rs770263622	E/D	280	0.758
68	rs749683576	C/R	285	0.925
69	rs1017054259	S/L	287	0.89
70	rs147498556	V/E	288	0.931
71	rs148560500	L/R	289	0.956
72	rs1487143659	L/S	292	0.892
73	rs200010345	I/T	299	0.866
74	rs867154905	I/N	302	0.934
75	rs944978839	P/S	311	0.875
76	rs74865777	M/V	321	0.914
77	rs200102110	V/L	324	0.796
78	rs867013198	T/I	331	0.9
79	rs1280180578	R/C	340	0.805
80	rs570647862	R/C	353	0.791
81	rs79109919	L/Q	363	0.725
82	rs1596064293	R/G	367	0.893
83	rs754450688	D/G	419	0.863
84	rs1327013276	W/C	420	0.916
85	rs150329151	V/F	426	0.938
86	rs138253116	R/W	429	0.942
87	rs368612437	R/Q	429	0.904
88	rs776726741	W/G	433	0.893
89	rs1266274639	F/L	435	0.876
90	rs759802002	F/C	435	0.924
91	rs202066018	L/R	436	0.89
92	rs148376112	G/R	442	0.925
93	rs200384194	S/Y	443	0.862
94	rs532896027	L/P	446	0.966
